# Supplementary material for: Antioxidant and Anti-Inflammatory Effect of Cinnamon (Cinnamomum verum J. Presl) Bark Extract after In Vitro Digestion Simulation
Source: Foods. 2023 Jan 18;12(3):452. doi: 10.3390/foods12030452 (PMC9914695; doi:10.3390/foods12030452)
Supplement: Supplementary file 1 [file foods-12-00452-s001.zip › foods-2131584-supplementary.pdf]

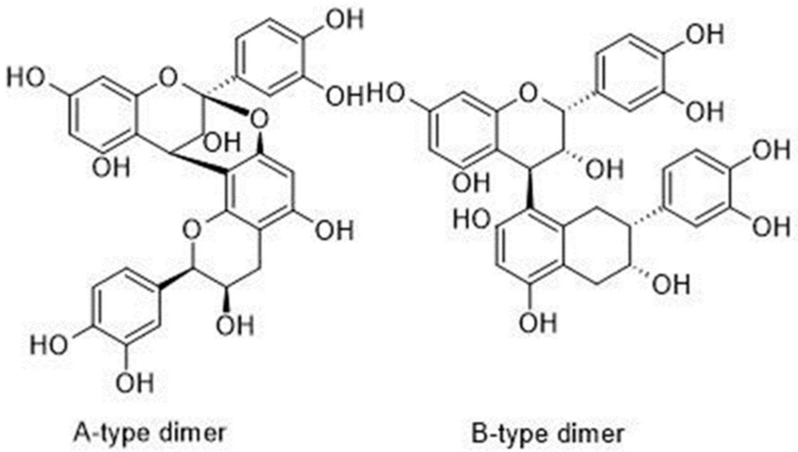

Figure S1. Procyanidins dimer A-type and B-type.

Table S1. Identification and quantification of metabolites in aqueous extract before and after digestion using mass spectrometry analysis by UHPLC-DAD-HESI-HRMS. In Table S1 are reported: peak number, probable identification, number of bonds for procyanidin species, retention time, chemical formula, theoretical and observed high-resolution m/z values, MS/MS fragments, and quantification (mg/ml). (* quantified as trans cinnamic acid; ** quantified as catechin.).

N°	Name	Number of bonds	Rt (min)	Formula	Mw [M-H]- Th	Mw [M-H]- Obs	Mw [M+H]+ Obs	MS/MS	(ug/10mgEXT) BEFORE DIGESTION λ=280 nm	(ug/10mgEXT) AFTER DIGESTION λ=280 nm	Quantification	Ref
1	Gallic acid		6.46	C7H6O5	169.014	169.014	-	125.024 - 169.014 - 69.035	361.48	< LOQ	*	A. Vallverdu-Queralt et al. (2014)

N°	Name	Number of bonds	Rt (min)	Formula	Mw [M-H]- Th	Mw [M-H]- Obs	Mw [M+H]+ Obs	MS/MS	(ug/10mgEXT) BEFORE DIGESTION λ=280 nm	(ug/10mgEXT) AFTER DIGESTION λ=280 nm	Quantification	Ref
2	Protocatechuic acid 4-glucoside		10.93	C13H16O9	315.072	315.072	-	108.022 - 315.072 - 153.011	2720.82	150.70	*	A. Akhtar et al. (2021)
3	3,4-Dihydroxybenzaldehyde		14.02	C7H6O3	137.024	137.024	-	-	2152.22	609.81	*	B. Klejdus et al. (2016)
4	Catechin		17.94	C15H14O6	289.072	289.072	281.086	289.071 - 245.082 - 261.890	1656.12	< LOQ	**	A. Vallverdu-Queralt et al. (2014)
5	4-Hydroxybenzaldehyd		18,37	C7H6O2	121,028	121,028	-	-	696.18	1071.2	*	A. Akhtar et al. (2021)
6	2-hydroxy-2-phenylacetic acid		22.20	C8H8O3	151.040	151.040	153.051	-	1615.18	664.1	*	A. Akhtar et al. (2021)

N°	Name	Number of bonds	Rt (min)	Formula	Mw [M-H]- Th	Mw [M-H]- Obs	Mw [M+H]+ Obs	MS/MS	(ug/10mgEXT) BEFORE DIGESTION λ=280 nm	(ug/10mgEXT) AFTER DIGESTION λ=280 nm	Quantification	Ref
7	Kaempferol o-C-di glucoside		22.52	C27H30O16	609.145	609.146	611.155	609.146 - 489.103 - 369.061	27641.57	1591.5	**	A. Vallverdu-Queralt et al. (2014)
8 a-b-c	Apigenin 6,8-di-C-glucoside		24.49 + 30.92 + 33.08	C27H30O15	593.151	593.151	595.167	593.151 - 353.066 - 383.077	37829.27	2258.43	**	A. Vallverdu-Queralt et al. (2014)
9	Coumaric acid		25.02	C9H8O3	163.040	163.040	-	-	1109.07	435.30	*	Standard
10	5,6,7,3',4'-Pentahydroxyisoflavone		28.43	C15H12O7	303.050	303.051	-	125.024 - 285.040 - 226.420	3216.81	< LOQ	**	A. Vallverdu-Queralt et al. (2014)
11	Coumarin		29.51	C9H6O2	147.044 [M+H]+	-	147.044	147.044 - 91.054 - 103.054	12739.64	7725.80	**	Standard
12	Apigenin 6-C-glucoside		29.76	C21H20O10	431.098	431.098	-	311.056 - 431.097 - 283.061	13846.88	842.4	**	A. Akhtar et al. (2021)

N°	Name	Number of bonds	Rt (min)	Formula	Mw [M-H]- Th	Mw [M-H]- Obs	Mw [M+H]+ Obs	MS/MS	(ug/10mgEXT) BEFORE DIGESTION λ=280 nm	(ug/10mgEXT) AFTER DIGESTION λ=280 nm	Quantification	Ref
13	Rutin		31.23	C27H30O16	609.145	609.145	611.155	609.145 - 301.035 - 271.025	1555.94	411.7	**	A. Vallverdu-Queralt et al. (2014)
14	Cinnamic acid derivate		32.10	C9H8O2	147.045	147.045	149.060	147.0450- 119.050 - 102.949	40453.96	3457.7	*	
15	Quercetin-3-O-rhamnoside		34.98	C21H20O11	447.093	447.093	449.107	300.027 - 301.035- 271.024	5511.44	303.3	**	A. Vallverdu-Queralt et al. (2014)
16	Trans-cinnamic acid		37.35	C9H8O2	147.045	147.045	149.060	147.045 - 119.050 - 102.949	40453.96	44463.7	*	B. Klejdus et al. (2016)
17	2-Hydroxybenzoic acid		14.37	C7H6O3	137.024	137.024	-	-	< LOQ	1174.2	*	A. Vallverdu-Queralt et al. (2014)
								**SUM (mg/Kg)**	**194722.6**	**66750.1**		

N°	Name	Number of bonds	Rt (min)	Formula	Mw [M-H]- Th	Mw [M-H]- Obs	Mw [M+H]+ Obs	MS/MS	(ug/10mgEXT) BEFORE DIGESTION λ=280 nm	(ug/10mgEXT) AFTER DIGESTION λ=280 nm	Quantification	Ref
18	Procyanidins dimer (Procyanidin B)	1	20.64	C30H26O12	577.134	577.135	579.149	289.071 - 407.077 - 125.024	20604.77	< LOQ	**	S. A. Lazarus et al. (1999) - N. Symma et al.(2021)
19 a	Procyanidins trimer (Cinnamtannin B1)	3	22.99	C45H36O18	863.182	863.182	865.197	411.071 - 289.071 - 711.135	279036.17	< LOQ	**	S. A. Lazarus et al (1999) - N. Symma (2021)
19 b	Procyanidins trimer (Cinnamtannin B1)	3	26.93	C45H36O18	863.182	863.182	865.97	411.071 - 289.071 - 711.135	17038.38	< LOQ	**	S. A. Lazarus et al (1999) - N. Symma (2021)
20 a	Procyanidins trimer	2	20.32	C45H38O18	865.197	865.199	867.212 - 889.194 [M+Na]+	125.024 - 407.077 - 289.071	8291.15	< LOQ	**	S. A. Lazarus et al (1999) - N. Symma (2021)
20 b	Procyanidins trimer	2	23.88	C45H38O18	865.197	865.199	867.212 - 889.194 [M+Na]+	125.024 - 407.077 - 289.071	13648.11	< LOQ	**	S. A. Lazarus et al (1999) - N. Symma (2021)

N°	Name	Number of bonds	Rt (min)	Formula	Mw [M-H]- Th	Mw [M-H]- Obs	Mw [M+H]+ Obs	MS/MS	(ug/10mgEXT) BEFORE DIGESTION λ=280 nm	(ug/10mgEXT) AFTER DIGESTION λ=280 nm	Quantification	Ref
20 c	Procyanidins trimer	2	25.34	C45H38O18	865.197	865.199	867.212 - 889.194[M+Na]+	125.024 - 407.077 - 289.071	3325.95	< LOQ	**	S. A. Lazarus et al (1999) - N. Symma (2021)
21 a	Procyanidins tetramer	3	18.40	C60H50O24	1153.261	1153.262 - 576.128 (Z=2)	1155.276	(MS/MS of Z =2) 125.024 -289.072 - 407.076	7151.75	< LOQ	**	S. A. Lazarus et al (1999) - N. Symma (2021)
21 b	Procyanidins tetramer	3	21.21	C60H50O24	1153.261	1153.262 - 576.128 (Z=2)	1155.276	(MS/MS of Z =2) 125.024 -289.072 - 407.076	75398.08	< LOQ	**	S. A. Lazarus et al (1999) - N. Symma (2021)
21 c	Procyanidins tetramer	3	21.64	C60H50O24	1153.261	1153.262 - 576.128 (Z=2)	1155.276	(MS/MS of Z =2) 125.024 -289.072 - 407.076	17971.63	< LOQ	**	S. A. Lazarus et al (1999) - N. Symma (2021)
21 d	Procyanidins tetramer	3	22.06	C60H50O24	1153.261	1153.262 - 576.128 (Z=2)	1155.276	(MS/MS of Z =2) 125.024 -289.072 - 407.076	9688.92	< LOQ	**	S. A. Lazarus et al (1999) - N. Symma (2021)
21 e	Procyanidins tetramer	3	24.92	C60H50O24	1153.261	1153.262 - 576.128 (Z=2)	1155.276	(MS/MS of Z =2) 125.024 -289.072 - 407.076	22295.69	< LOQ	**	S. A. Lazarus et al (1999) - N. Symma (2021)

N°	Name	Number of bonds	Rt (min)	Formula	Mw [M-H]- Th	Mw [M-H]- Obs	Mw [M+H]+ Obs	MS/MS	(ug/10mgEXT) BEFORE DIGESTION λ=280 nm	(ug/10mgEXT) AFTER DIGESTION λ=280 nm	Quantification	Ref
21 f	Procyanidins tetramer	3	25.20	C60H50O24	1153.261	1153.262 - 576.128 (Z=2)	1155.276	(MS/MS of Z =2) 125.024 -289.072 - 407.076	5684.91	< LOQ	**	S. A. Lazarus et al (1999) - N. Symma (2021)
21 g	Procyanidins tetramer	3	25.81	C60H50O24	1153.261	1153.262 - 576.128 (Z=2)	1155.276	(MS/MS of Z =2) 125.024 -289.072 - 407.076	11542.76	< LOQ	**	S. A. Lazarus et al (1999) - N. Symma (2021)
21 h	Procyanidins tetramer	3	3.39	C60H50O24	1153.261	1153.262 - 576.128 (Z=2)	1155.276	(MS/MS of Z =2) 125.024 -289.072 - 407.076	11803.23	< LOQ	**	S. A. Lazarus et al (1999) - N. Symma (2021)
22 a	Procyanidins tetramer	4	15.41	C60H48O24	1151.245	1151.245 - 575.119 (Z=2)	1153.260	411.071 - 573.103 - 863.182	31059.79	< LOQ	**	S. A. Lazarus et al (1999) - N. Symma (2021)
22 b	Procyanidins tetramer	4	16.00	C60H48O24	1151.245	1151.245 - 575.119 (Z=2)	1153.260	411.071 - 573.103 - 863.182	104744.28	< LOQ	**	S. A. Lazarus et al (1999) - N. Symma (2021)
22 c	Procyanidins tetramer	4	22.49	C60H48O24	1151.245	1151.245 - 575.119 (Z=2)	1153.260	411.071 - 573.103 - 863.182	22389.54	< LOQ	**	S. A. Lazarus et al (1999) - N. Symma (2021)

N°	Name	Number of bonds	Rt (min)	Formula	Mw [M-H]- Th	Mw [M-H]- Obs	Mw [M+H]+ Obs	MS/MS	(ug/10mgEXT) BEFORE DIGESTION λ=280 nm	(ug/10mgEXT) AFTER DIGESTION λ=280 nm	Quantification	Ref
22 d	Procyanidins tetramer	4	24.00	C60H48O24	1151.245	1151.245 - 575.119 (Z=2)	1153.260	411.071 - 573.103 - 863.182	17031.53	< LOQ	**	S. A. Lazarus et al (1999) - N. Symma (2021)
23 a	Procyanidins pentamer	4	21.64	C75H62O30	1441.324 (Z=2) 720.155	(Z=2) 720.159	-	289.071 - 407.077 - 449.087	28714.54	< LOQ	**	S. A. Lazarus et al (1999) - N. Symma (2021)
23 b	Procyanidins pentamer	4	22,82	C75H62O30	1441.324 (Z=2) 720.155	(Z=2) 720.159	-	289.071 - 407.077 - 449.087	8321.21	< LOQ	**	S. A. Lazarus et al (1999) - N. Symma (2021)
23 c	Procyanidins pentamer	4	25,17	C75H62O30	1441.324 (Z=2) 720.155	(Z=2) 720.159	-	289.071 - 407.077 - 449.087	7385.32	< LOQ	**	S. A. Lazarus et al (1999) - N. Symma (2021)
23 d	Procyanidins pentamer	4	26.11	C75H62O30	1441.324 (Z=2) 720.155	(Z=2) 720.159	-	289.071 - 407.077 - 449.087	14748.50	< LOQ	**	S. A. Lazarus et al (1999) - N. Symma (2021)
23 a	Procyanidins pentamer	5	17.60	C75H60O30	1439.310 (Z=2) 719.151	(Z=2) 719.151	1441.323 - (Z=2) 721.165	289.072 - 411.071 - 451.103	4980.49	< LOQ	**	S. A. Lazarus et al (1999) - N. Symma (2021)

N°	Name	Number of bonds	Rt (min)	Formula	Mw [M-H]- Th	Mw [M-H]- Obs	Mw [M+H]+ Obs	MS/MS	(ug/10mgEXT) BEFORE DIGESTION λ=280 nm	(ug/10mgEXT) AFTER DIGESTION λ=280 nm	Quantification	Ref
24 b	Procyanidins pentamer	5	18.58	C75H60O30	1439.310 (Z=2) 719.151	(Z=2) 719.151	1441.323 - (Z=2) 721.165	289.072 - 411.071 - 451.103	25881.05	< LOQ	**	S. A. Lazarus et al (1999) - N. Symma (2021)
24 c	Procyanidins pentamer	5	19.44	C75H60O30	1439.310 (Z=2) 719.151	(Z=2) 719.151	1441.323 - (Z=2) 721.165	289.072 - 411.071 - 451.103	38777.81	< LOQ	**	S. A. Lazarus et al (1999) - N. Symma (2021)
24 d	Procyanidins pentamer	5	20.26	C75H60O30	1439.310 (Z=2) 719.151	(Z=2) 719.151	1441.323 - (Z=2) 721.165	289.072 - 411.071 - 451.103	15731.31	< LOQ	**	S. A. Lazarus et al (1999) - N. Symma (2021)
24 e	Procyanidins pentamer	5	23.56	C75H60O30	1439.310 (Z=2) 719.151	(Z=2) 719.151	1441.323 - (Z=2) 721.165	289.072 - 411.071 - 451.103	19934.62	< LOQ	**	S. A. Lazarus et al (1999) - N. Symma (2021)
24 f	Procyanidins pentamer	5	27.38	C75H60O30	1439.310 (Z=2) 719.151	(Z=2) 719.151	1441.323 - (Z=2) 721.165	289.072 - 411.071 - 451.103	5073.29	< LOQ	**	S. A. Lazarus et al (1999) - N. Symma (2021)
24 g	Procyanidins pentamer	5	28.02	C75H60O30	1439.310 (Z=2) 719.151	(Z=2) 719.151	1441.323 - (Z=2) 721.165	289.072 - 411.071 - 451.103	7583.04	< LOQ	**	S. A. Lazarus et al (1999) - N. Symma (2021)

N°	Name	Number of bonds	Rt (min)	Formula	Mw [M-H]- Th	Mw [M-H]- Obs	Mw [M+H]+ Obs	MS/MS	(ug/10mgEXT) BEFORE DIGESTION λ=280 nm	(ug/10mgEXT) AFTER DIGESTION λ=280 nm	Quantification	Ref
25 a	Procyanidins hexamer	6	21.07	C90H72O36	1727.373 (Z=2) 863.183	(Z=2) 863.183	865,197	289.071 - 411.071 - 573.103	7593.59	< LOQ	**	S. A. Lazarus et al (1999) - N. Symma (2021)
25 b	Procyanidins hexamer	6	21.54	C90H72O36	1727.373 (Z=2) 863.183	(Z=2) 863.183	865.197	289.071 - 411.071 - 573.103	5886.32	< LOQ	**	S. A. Lazarus et al (1999) - N. Symma (2021)
25 c	Procyanidins hexamer	6	22.15	C90H72O36	1727.373 (Z=2) 863.183	(Z=2) 863.183	865.197	289.071 - 411.071 - 573.103	15041.65	< LOQ	**	S. A. Lazarus et al (1999) - N. Symma (2021)
25 d	Procyanidins hexamer	6	24.48	C90H72O36	1727.373 (Z=2) 863.183	(Z=2) 863.183	865.197	289.071 - 411.071 - 573.103	29339.87	< LOQ	**	S. A. Lazarus et al (1999) - N. Symma (2021)
26	Procyanidins heptamer	6	broad peak	C105H86O42	(Z=2) 1008.724	(Z=2) 1008.724	(Z=2) 1010.738	289.071 - 411.071 - 573.103	133494.67	< LOQ	**	S. A. Lazarus et al (1999) - N. Symma (2021)
	Procyanidins heptamer	7		C105H84O42	(Z=2) 1007.716	(Z=2) 1007.717	(Z=2) 1009.730	289.071 - 411.071 - 573.103				S. A. Lazarus et al (1999) - N. Symma (2021)

N°	Name	Number of bonds	Rt (min)	Formula	Mw [M-H]- Th	Mw [M-H]- Obs	Mw [M+H]+ Obs	MS/MS	(ug/10mgEXT) BEFORE DIGESTION λ=280 nm	(ug/10mgEXT) AFTER DIGESTION λ=280 nm	Quantification	Ref
	Procyanidins octamer	7		C120H98O48	(Z=2) 1152.756	(Z=2) 1152.755	-	1152.249 - 412.075 - 574.108				S. A. Lazarus et al (1999) - N. Symma (2021)
	Procyanidins octamer	8		C120H96O48	(Z=2) 1151.246	(Z=2) 1151.246	(Z=2) 1153.762	411.072 - 285.041 - 573.103				S. A. Lazarus et al (1999) - N. Symma (2021)
								**SUM (mg/Kg)**	**1047193.9**	**< LOQ**		

**TOTAL (mg/Kg)**	**1241916.5**	**66750.1**
